# Comparison of Emergency Physicians’ and Hospitalists’ Attitudes Toward Fecal Occult Blood Testing in Gastrointestinal Bleeding

**DOI:** 10.5811/westjem.47193

**Published:** 2025-11-26

**Authors:** Doris Ilic, Joseph Bove

**Affiliations:** Saint Joseph’s University Medical Center, Department of Emergency Medicine, Paterson, New Jersey

## Abstract

**Introduction:**

The guaiac fecal occult blood test, originally designed for colorectal cancer screening, is frequently used in emergency departments (ED) to detect occult gastrointestinal (GI) bleeding. However, the test has low sensitivity and specificity, leading to potential false positives and negatives. This study evaluates the current practices and perceptions of emergency physicians and hospitalists regarding the utility of the guaiac test in the setting of suspected GI bleeding in the ED.

**Objective:**

Our primary aim in this study was to evaluate the current practice and views of emergency physicians and hospitalists on the utility of the stool guaiac test in the ED.

**Methods:**

We conducted a multicenter survey from January 3–April 3, 2024, across four hospital systems, targeting attending physicians in the ED and hospitalists. Participants were asked to rate their agreement with statements about the stool guaiac test on a scale of 1 (strongly disagree) to 5 (strongly agree).

**Results:**

Response rates were 47/93 (50.5%) for emergency attendings and 9/18 (50%) for hospitalists. Emergency attendings were significantly less likely than hospitalists to agree that stool guaiac testing is important for evaluating GI bleeding (31% vs 67%, P < .001). More than half of emergency attendings (55%) reported often performing the test, while 44% of hospitalists reported frequently requesting it before accepting a patient. Although 70% of emergency attendings believed that guaiac results influence hospitalists’ admission decisions (P = .02), 67% of hospitalists stated they would accept a patient with suspected GI bleeding even without a result. Despite rating the test as important, only 33% of hospitalists felt that stool guaiac testing frequently changes management during hospitalization. Overall, the groups showed distinct attitudes regarding the utility and impact of stool guaiac testing.

**Conclusion:**

The guaiac fecal occult blood test remains widely used despite skepticism among emergency attendings regarding its importance. Hospitalists were more likely to request the test but acknowledged it rarely changes patient management. These findings highlight the need for re-evaluation of guaiac testing in acute care settings and improved communication between ED and inpatient teams. Further research should explore the clinical impact of removing routine stool guaiac testing.

## INTRODUCTION

Guaiac-based fecal occult blood tests were initially developed and approved as a screening tool for colorectal cancer.^1^ However, in the emergency department (ED), they are frequently used to assess for occult blood in the stool when evaluating patients with suspected gastrointestinal (GI) bleeding. Despite their widespread use, guaiac tests have significant limitations in this context. A 2024 study analyzing hospitalized patients with suspected GI bleeding found that guaiac-based fecal occult blood tests were frequently used inappropriately and rarely contributed to changes in management, highlighting its limited clinical utility in the inpatient setting.^2^ Additionally, a 2020 systematic review and meta-analysis found that guaiac-based fecal occult blood tests had a pooled sensitivity of 58% and specificity of 84% for detecting GI sources of iron deficiency anemia, underscoring the potential for both false negatives and false positives.^3^

Studies have also demonstrated high false-positive rates in the ED, often due to dietary factors, medications, or other non-GI sources of blood that are difficult to control for in emergent situations.^4^ Furthermore, false negatives can occur, particularly in cases of intermittent or slow bleeding, further complicating clinical decision-making. A retrospective chart review similarly concluded that the guaiac-based fecal occult blood test is not useful as a diagnostic test in symptomatic patients, reinforcing concerns about its limited utility in acute care settings.^1^

Our primary aim in this study was to evaluate the current practice patterns and perceptions of physicians regarding the utility of stool guaiac testing. We surveyed both emergency physicians and inpatient hospitalists to explore their reliance on this test for patients presenting with suspected GI bleeding in the ED. We hypothesized that despite its limitations, stool guaiac testing remains a widely used and valued tool among emergency physicians and that hospitalists continue to request the test upon patient admission.

## METHODS

This was a multicenter, survey-based study involving four hospital systems. Our institution is a large, urban, tertiary-care center; the other hospitals were Mount Sinai Miami, Jackson North Medical Center, AdventHealth Kissimmee, and Newark Beth Israel. To collect information for this study, we used a Google survey (Google. LLC, Mountain View, CA) from January 3–April 3, 2024. One survey was sent specifically to emergency attending physicians while one survey was sent only to hospitalists. A hospitalist, for the purpose of our study, was defined as a physician who admits patients to the inpatient floors. Critical care physicians were not surveyed nor was any physician who practiced solely outpatient. Exclusion criterion was an incomplete survey. On both surveys, we collected the respondent’s number of years post residency training as well as the name of the hospital in which the participant practiced.

Survey respondents’ identities remained anonymous, as the survey was designed to avoid collecting any personally identifiable information. Participants were then asked a series of questions and were asked to choose an answer between 1–5, with 1 being strongly disagree and 5 corresponding to strongly agree.

### Emergency Attending Survey Questions

1. It is very important to do a guaiac test in the evaluation of a GI bleed.
2. I often do a guaiac test on a patient with suspicion of GI bleeding in the ED.
3. Guaiac test results frequently change the management or disposition of my patient.
4. The admitting team is often influenced by a guaiac result.

**Population Health Research Capsule**

What do we already know about this issue?

*Stool guaiac testing is used in the ED for gastrointestinal (GI) bleeding, despite concerns about poor sensitivity, specificity, and limited impact on clinical management.*

What was the research question?

*How do emergency physicians’ and hospitalists’ attitudes differ toward guaiac testing for GI bleeding?*

What was the major finding of the study?

*33% of emergency attendings vs 67% of hospitalists valued testing (absolute difference 34 %, 95% CI 0.5*–*67, P = .02)*

How does this improve population health?

*This study highlights the need to re-evaluate routine guaiac testing in acute care, aligning practice with evidence to reduce unnecessary testing and improve care.*

### Hospitalist Survey Questions

1. Obtaining a guaiac test is important in the evaluation of a GI bleed.
2. I would accept a patient that presents with signs of GI bleed without a guaiac test result.
3. I often ask for a guaiac test before accepting a patient for admission for suspected GI bleed.
4. Guaiac test results frequently change the management of my patient throughout their hospital course.

### Data Analysis

We conducted all statistical analyses using Python (Python Software Foundation, Wilmington, DE). Descriptive statistics were used to summarize survey responses. We compared categorical responses between emergency attendings and hospitalists using chi-square tests or Fisher exact tests, where appropriate. A two-tailed *P*-value of < .05 was considered statistically significant.

## RESULTS

We received 47 responses from emergency physician attendings (50.5% response rate) and nine responses from hospitalists (50% response rate), of a total 93 emergency attendings and 18 hospitalists who were sent surveys. When comparing the two groups, 31% of emergency attendings agreed or strongly agreed that the stool guaiac test is important for GI bleed evaluation, compared to 67% of hospitalists. Slightly more than half (55%) of emergency physician attendings reported that they often perform the test on patients with suspected GI bleeding, while 44% of hospitalists reported that they often ask for a stool guaiac test before accepting a patient for admission. Additionally, 70% of emergency attendings agreed or strongly agreed that the admitting team is influenced by guaiac test results, compared to only 33% of hospitalists who felt that the test frequently changes management throughout a patient’s hospital course. Despite the perceived importance, 67% of hospitalists agreed or strongly agreed that they would accept a patient with signs and symptoms of GI bleeding even without a stool guaiac test result.

Although only some group differences reached statistical significance—specifically, the emergency physician attendings’ lower valuation of the test’s importance (*P* < .001) and their perception of its influence on admission decisions (*P* = .02)—the overall trends highlight notable differences in attitudes between emergency attendings and hospitalists regarding the use of stool guaiac testing in clinical decision-making.

**Figure 1:**
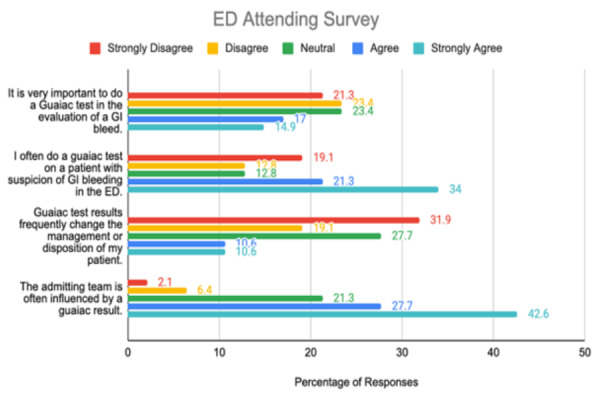
Attitudes of attending emergency physicians toward guaiac fecal occult blood testing for gastrointestinal bleeding based on a multicenter survey. *ED*, emergency department; *GI*, gastrointestinal.

The Figure 1 bar chart displays the percentage of responses to four Likert-scale items assessing emergency attendings’ perceptions and practices regarding guaiac fecal occult blood testing in patients with suspected GI bleeding. The survey was distributed across four hospital systems.

**Figure 2:**
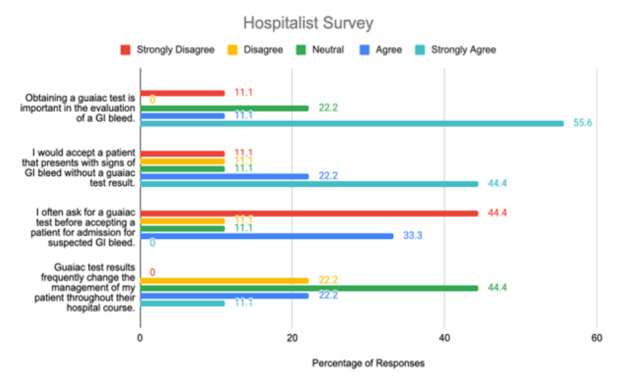
Attitudes of hospitalists toward guaiac fecal occult blood testing for gastrointestinal bleeding based on a multicenter survey. *GI*, gastrointestinal.

Figure 2 presents the distribution of hospitalist responses to four Likert-scale survey questions evaluating their views on the role of stool guaiac testing in patients presenting with suspected GI bleeding. The questions address test importance, its necessity for admission, likelihood of requesting it before admission, and whether it influences patient management. Results are shown as percentages across agreement levels, allowing comparison of hospitalists’ perspectives on the clinical utility of the test.

## DISCUSSION

We sought to evaluate the current practices and perceptions of emergency physicians and hospitalists regarding the utility of the stool guaiac test in patients with suspected GI bleeding in the ED. The findings of our study resonate with the ongoing debate in the literature regarding the utility of guaiac testing in GI bleeding. Previous research has highlighted the significant limitations of stool guaiac testing, including its low sensitivity and specificity, especially in the emergency setting where dietary and medication restrictions cannot be controlled.^1^ Despite these limitations, our results show a continued reliance on this test, reflecting ingrained practice patterns and complex decision-making dynamics in acute care settings.

The survey results revealed a complex perception of stool guaiac testing among emergency attendings. While 70% agreed or strongly agreed that guaiac test results influenced admitting team decisions (*P* = .02), only 31% felt the test was important for GI bleed evaluation (*P* < .001). This dichotomy suggests that while emergency physicians may not place high diagnostic value on the test, they recognize its impact on interdepartmental communication and disposition decisions; 34% of emergency attendings still frequently performed the test, suggesting that its use persists, potentially due to ingrained practice habits or the perceived expectations of inpatient teams. In contrast, hospitalists demonstrated a stronger inclination toward stool guaiac testing, with 67% agreeing on its importance, although this was not statistically significant (*P* = .51). Interestingly, 67% of hospitalists indicated they would accept a patient with suspected GI bleeding without a guaiac test result, highlighting a pragmatic approach that aligns with literature advising against overreliance on the test.^4^ The discrepancy between perceived importance and willingness to admit without a result suggests hospitalists may view stool guaiac testing as part of a broader clinical picture rather than a decisive factor in management.

The persistent use of stool guaiac testing in both emergency and inpatient settings highlights a disconnect between clinical practice and evidence-based recommendations. Despite well-documented limitations, many clinicians continue to rely on guaiac fecal occult blood testing in the evaluation of GI bleeding. Research consistently cautions against the routine use of this test in symptomatic patients due to its high rates of false positives and false negatives.^2^ Some institutions have recognized these shortcomings and have taken steps to eliminate in-hospital fecal occult blood testing altogether. Notably, these initiatives have often been spearheaded by gastroenterologists advocating for practice changes based on the test’s poor diagnostic accuracy and limited clinical utility.^5^ The push to discontinue routine use reflects a growing awareness that the risks of false reassurance, unnecessary testing, and potential delays in care outweigh any perceived benefits of stool guaiac testing in acute settings.

One of the major flaws of stool guaiac testing is its susceptibility to external factors, leading to unreliable results. Certain foods and medications can cause false positives, further complicating its utility in emergent evaluations. For instance, foods such as red meat, turnips, broccoli, cauliflower, and radishes have been shown to trigger false-positive results. Additionally, medications including nonsteroidal anti-inflammatory drugs and anticoagulants are known to interfere with test outcomes.^1^ Given the unpredictable nature of ED presentations, it is impractical to expect patients to adhere to the strict dietary and medication restrictions required for accurate test interpretation. This inherent limitation makes the test unreliable for guiding real-time clinical decision-making. The high false-positive rate can lead to a cascade of unnecessary interventions, including endoscopic procedures, excessive resource utilization, and prolonged hospitalizations, all of which contribute to increased healthcare costs and potential patient harm.

Beyond its susceptibility to false positives, stool guaiac testing is also limited by its poor sensitivity in detecting significant GI pathology. A 2020 systematic review and meta-analysis analyzing 22 studies found that guaiac fecal occult blood testing had poor sensitivity for detecting iron deficiency anemia due to GI blood loss. The study revealed that 42% of patients with identifiable causes of iron deficiency anemia had false-negative fecal occult blood testing results, emphasizing the test’s inability to reliably detect GI bleeding.^3^ Another study found that over 25% of patients with overt GI bleeding experienced delays in specialist referral because clinicians were waiting for fecal occult blood testing results before proceeding with further evaluation.^1^ These findings illustrate that inappropriate use of this test has the potential to delay necessary patient care. Despite these limitations, we found that more than 30% of hospitalists still believe that stool guaiac testing frequently influences patient management, underscoring the persistence of outdated practice patterns.

The most recent survey-based study assessing the use of stool guaiac testing in the ED was conducted in 2020.^6^ This study sought to evaluate whether emergency physicians could accurately predict the results of guiac-based fecal occult blood tests and whether the test influenced patient disposition. Interestingly, the survey found that emergency physicians were unable to consistently predict test results, which some interpreted as a potential justification for using the guaiac-based fecal occult blood test as a confirmatory tool in clinical evaluation. However, the study did not assess actual patient outcomes, and given the test’s known low sensitivity and specificity, its unpredictability only further reinforces its lack of clinical value.^6^

Our findings align with this conclusion, as most of the emergency physicians in our study reported using the guaiac-based fecal occult blood test primarily to reinforce clinical impressions for the admitting team rather than as a decisive diagnostic tool. Notably, the 2020 survey also revealed that emergency physicians were more likely to anticipate that guaiac-based fecal occult blood test results would alter patient disposition before performing the test, but this likelihood decreased after the test was completed.^6^ This suggests that clinicians initially expect the guaiac-based fecal occult blood test to provide useful information, only to find that the results rarely impact their clinical decision-making in a meaningful way.

These findings collectively highlight the need for a reassessment of the role of stool guaiac testing in acute care settings. Although historical practice may have supported its use, increasing evidence indicates that discontinuing routine guaiac-based fecal occult blood testing in symptomatic patients has the potential to streamline patient care, reduce unnecessary interventions and costs, and promote adherence to evidence-based practice; however, our study was not designed to demonstrate this effect.

## LIMITATIONS

Our study’s findings are limited by the overall small response rates from both hospitalists and emergency attendings, which constrains our ability to generalize the results across inpatient and ED settings. Additionally, as participation was voluntary, there is potential for selection bias, as those with stronger opinions on stool guaiac testing may have been more likely to complete the survey. However, previous research suggests that low response rates do not inherently compromise the validity or reliability of survey-based findings if the responding sample is reasonably representative of the target population.^7^–^9^ Lastly, our study evaluates physician perceptions and self-reported practice patterns but does not assess actual patient outcomes or the direct clinical impact of stool guaiac testing on management decisions.

The results suggest a need for re-evaluation of the routine use of stool guaiac testing in the ED, with a focus on enhancing education and communication between emergency physicians and hospitalists regarding its limitations. Future research assessing the impact of its removal by comparing clinical practices and outcomes before and after discontinuation could provide valuable insights.

## CONCLUSION

The guaiac-based fecal occult blood test remains widely used in emergency departments, despite skepticism among emergency attendings regarding its clinical value. Hospitalists were more likely to request the test, although they acknowledged it infrequently changes patient management. These findings suggest a need to re-evaluate the routine use of guaiac testing in acute care settings and to strengthen communication between ED and inpatient teams about its role in clinical decision-making. Future research should focus on outcomes associated with the removal of routine stool guaiac testing to better align practice with the available evidence.
